# Management of BNT162b2 mRNA COVID-19 vaccine in children aged 5–11 years with allergies, asthma, and immunodeficiency: consensus of the Italian Society of Pediatric Allergy and Immunology (SIAIP)

**DOI:** 10.1186/s13052-022-01272-z

**Published:** 2022-05-16

**Authors:** Elio Novembre, Mariangela Tosca, Carlo Caffarelli, Mauro Calvani, Fabio Cardinale, Riccardo Castagnoli, Elena Chiappini, Claudio Cravidi, Michele Miraglia Del Giudice, Marzia Duse, Amelia Licari, Sara Manti, Alberto Martelli, Giampaolo Ricci, Giuseppe Pingitore, Gian Luigi Marseglia

**Affiliations:** 1 Allergy Unit, Department of Pediatrics, Meyer Children’s University Hospital, Florence, Italy; 2 Allergy Center, Istituto Giannina Gaslini, Genoa, Italy; 3 Pediatric Clinic, Department of Medicine and Surgery, University of Parma, Via Gramsci, 14 Parma, Italy; 4 Operative Unit of Pediatrics, S. Camillo-Forlanini Hospital, Rome, Italy; 5 Pediatric Unit, Azienda Ospedaliero-Universitaria “Policlinico- Giovanni XXIII, Bari, Italy; 6 Pediatric Clinic, Fondazione IRCCS Policlinico San Matteo, University of Pavia, Pavia, Italy; 7 Department of Clinical, Surgical, Diagnostic and Pediatric Sciences, University of Pavia, Pavia, Italy; 8 Paediatric Infectious Diseases Unit, Department of Health Sciences, Anna Meyer Children’s University Hospital, University of Florence, Florence, Italy; 9 ATS Pavia, Pavia, Italy; 10 Department of Woman and Child and General and Specialized Surgery, University of Campania Luigi Vanvitelli, Naples, Italy; 11 Department of Maternal Infantile and Urological Sciences, Sapienza University of Rome, Rome, Italy; 12 Pediatric Respiratory Unit, Department of Clinical and Experimental Medicine, San Marco Hospital, University of Catania, Catania, Italy; 13 Pediatrician at the University of Pavia, Pavia, Italy; 14 Alma Mater Studiorum, Department of Medical and Surgical Sciences, University of Bologna, Bologna, Italy; 15 Pediatrician and Allergist, Rome, Italy

**Keywords:** COVID-19, SARS-CoV-2, Vaccine, Children, Adverse event, Allergy, Side effect, mRNA vaccine, Pfizer BioNtech, BNT162b2

## Abstract

BNT162b2 vaccine, developed by BioNTech and Pfizer ha recently approved for use in children aged 5 to 11 years. Recent data show evidence of safety on the administration and serious adverse events have been rarely reported. However, allergic systemic reactions could occur. In some cases, a correct allergic evaluation allows identifying patients at risk of developing an anaphylactic reaction. Risk assessment of allergic reactions to COVID-19 vaccines is useful to limit contraindications to vaccination and help to safely vaccinate people supposed to be at risk of allergic reactions.

## Introduction

The Committee for Medicinal Products for Human Use of the European Medicines Agency [1] and AIFA (Italian Medicines Agency) have recently approved the extension of the indication to the BNT162b2 vaccine, developed by BioNTech and Pfizer, for use in children aged 5 to 11 years [2]. The Italian Ministry of Health (Circular 56,429, 7 December 2021) has recently confirmed this extended indication. The BNT162b2 vaccine dose is lower than that used in people 12 years of age and older (10 μg vs. 30 μg). As in the older age group, it is administered intramuscularly in the upper arm and repeated 3 weeks later. The vaccination for children 5–11 years-old is less concentrated and contains excipients such as polyethylene glycol (PEG) and trometamol, but not polysorbates. Therefore, an allergic reaction could occur for one of these excipients, although allergic reactions to trometamol are exceptionally reported [3]. Clinical data on the BNT162b2 vaccine in children aged 5 to 11 years are reported by Centers for Disease Control and Prevention [4]. The most common side effects in this age group are similar to those observed in people aged 12 and over. These data show evidence of safety on the administration of BNT162b2 vaccine in children aged 5 to 11 years and serious adverse events (mainly myocarditis) have been rarely reported. However, parents and guardians of children aged 5 to 11 years should be advised that after BNT162b2 vaccine, local and, rarely, systemic reactions could occur, more often after the second dose. A drug/vaccine allergy history is not a contraindication for the BNT162b2 vaccination unless the offending product contains the same excipients. An IgE-dependent allergy is due to antibody recognition of a chemical structure. In this context, a correct evaluation of the allergic response allows identifying patients at real risk of developing an anaphylactic reaction in case of further exposure. Several international public health agencies and allergy organizations worldwide have published guidance related to the concerns for possible severe allergic reactions, specifically to the mRNA vaccine excipients. All reports suggest, in suspected cases, to refer to an allergist for assessment [5–14].

### Risk stratification in allergic and asthmatic children

On the basis of international documents [5–14], recommendations for adolescents 12–18 years-old of the Italian Society of Pediatric Allergy and Immunology (SIAIP) [15], Italian documents from the Italian Society of Allergy and Clinical Immunology (SIAIC), the Association of Italian Territorial Hospital Allergists (AAITO) [16], and the Italian Ministry of Health [17], three “risk zones” of possible allergic reactions could be identified (Table 1)

1. *Low risk (Green zone)*
  This zone includes patients with allergic rhinoconjunctivitis, well-controlled asthma, and non-anaphylactic allergic reactions to foods, insects, and latex.
  Action: Proceed with vaccination as usual (as in non-allergic subjects), according to local guidelines. In children treated with allergen immunotherapy, COVID-19 vaccines should be administered at the interval of 7 days from subcutaneous immunotherapy Likewise, sublingual daily dose should be stopped 3 days before COVID-19 vaccine administration and restarted 7 days after [18].
2. *Medium risk (Yellow zone)*
  This zone includes the following clinical pictures.
  History of anaphylactic reactions to foods, insects, latex.
  The following definition of anaphylaxis was considered in these cases: “Anaphylaxis is a serious systemic hypersensitivity reaction that is usually rapid in onset and may cause death. Severe anaphylaxis is characterized by potentially life-threatening compromise in breathing and/or the circulation, and may occur without typical skin features or cardio-circulatory shock being present” [19].
  Action: If the cause of anaphylaxis is confirmed, COVID-19 vaccination can be performed. Most international documents suggest [7, 9–11, 20] to proceed with vaccination as normal (in the green zone). However, considering that some patients who developed allergic reactions after BNT162b2 vaccine have a previous history of food anaphylaxis [21–23] we suggest performing the vaccine in vaccination centers equipped for anaphylaxis management [6, 24].
  Idiopathic anaphylaxis and exercise-induced anaphylaxis.
  Action: Proceed with the vaccination (in centers and facilities equipped for anaphylaxis management or in the hospital setting, according to teleconsulting or diagnostic workup [14, 16]**.**
  Mastocytosis**.**
    COVID-19 vaccination is generally recommended in patients with mastocytosis. Safety measures, including pharmacological pre-medication and post-vaccination observation, should be considered in all patients with mastocytosis, depending on the individual risk and general conditions of every single case [16, 25].
    Action: In most cases, proceed with routine vaccination in an outpatient setting with emergency awareness and emergency medication available (24). Pre-medication with sedating or non-sedating H1 antihistamine and supervision for 60 minutes after vaccination should be considered.
    For the high-risk population (previous anaphylaxis, also to a vaccine, known or suspected allergy to excipients, systemic mastocytosis): see red zone.
  Uncontrolled asthma.
    According to GINA document [26], asthma in children is not controlled if the child (or parents) answers “yes” at least to 3 of these questions: i) Presence of daytime asthma symptoms more than twice/week?; ii) Is there any night waking due to asthma?; iii) Use of Short-Acting Beta-Agonists (SABA) reliever for symptoms more than twice/week?; iv) Is there any activity limitation due to asthma?
    Action: Try to reach the best possible asthma control and then proceed to vaccination. If asthma is severe or only partially controlled, proceed with vaccination in a hospital setting with an observation of at least 60 minutes [16, 27]. COVID-19 vaccine should be administered 2–7 days after biologic administration in asthmatic patients treated with biologics [18].
  Large local reaction to previous COVID-19 vaccination.
    Action: some documents [11, 14, 28] include this reaction in the green zone. However, according to other consensus (8), we suggest to perform teleconsulting and eventually specific risk assessment in some cases of immediate reaction, in order to exclude a possible allergy to PEG or another excipient. The general indication is to perform vaccination in an outpatient setting with emergency awareness and emergency medication available. Pre-medication with H1 antihistamine should be considered.

- Immediate allergic reactions to drugs or vaccines.
  Action: specific risk assessment concerning a possible PEG (or another excipient) allergy is required [14, 16, 29]. If skin tests result negative, vaccination should be performed in a hospital setting with an observation of at least 60 minutes. If skin tests result positive, refer to the red zone.
  Generally, allergy assessment or teleconsulting are indicated in all above cases, if requested by pediatricians or vaccination centers. According to the allergy assessment or teleconsulting, vaccination should be performed in settings where symptoms of anaphylaxis can be recognized as early as possible and promptly treated with epinephrine and further emergency treatment or in hospital settings where it is possible to continue monitoring/treating of the patient in case of need [13, 16, 17, 27]. The allergy assessment or teleconsulting aims to make a certain diagnosis and give specific indications: modify therapy in case of uncontrolled or partially controlled asthma, or pharmacological pre-medication.

3) *High risk (Red zone)*
  This zone includes patients with the following:
  Positive skin tests to excipients
  Previous allergic reaction to the specific vaccine
  Previous severe allergic reaction to a vaccine component or drugs, including PEG or trometamol
    Action: perform allergy risk assessment; consider that sensitivity and specificity for a skin test to excipients is unknown, and the diagnostic utility of PEG and polysorbate allergy testing is uncertain [5]. According to skin test results, the patient may be considered ineligible for a further vaccination or eligible for another vaccination containing another excipient negative to the skin test. Vaccination in fractionated doses could be considered in selected cases [14]. In all these cases, vaccination must be carried out under strict control in a hospital setting where emergency medical services for resuscitation are available [9, 16, 28].

**Table 1:** Allergic risk assessment for COVID-19 vaccination in children

**Low risk**	
**Patient characteristics**	**Action**
Allergic rhinoconjunctivitis and allergic well-controlled asthma Non-anaphylactic allergic reactions to food, insects, and latex	Proceed with vaccinations as usual, according to local guidelines. No allergic evaluation is needed. Therapies for allergies and/or asthma must be continued as usual. Children treated with allergen immunotherapy (AIT) should withhold administration for few days (see text).
**Medium risk**	
**Patient characteristics**	**Action**
Anaphylactic allergic reactions to food, insects, and latex	Consider referral (or teleconsulting) to an allergist-immunologist to confirm the diagnosis and give proper indications. Reach the best possible asthma control and then proceed to vaccination. If asthma control is suboptimal, proceed to vaccination in a hospital setting with an observation of at least 60 minutes.
Idiopathic anaphylaxis and exercise-induced anaphylaxis	
Uncontrolled asthma	
Mastocytosis	Routine vaccination in an outpatient setting with emergency awareness and emergency medication available. Pre-medication with H1 antihistamine should be considered. For high-risk population (previous anaphylaxis, also to vaccinations, known or suspected allergy to excipients, systemic mastocytosis) see red zone.
Large local reaction to previous COVID-19 vaccination	Specific risk assessment (or teleconsulting) to exclude a possible allergy to PEG or another excipient. Routine vaccination in an outpatient setting with emergency awareness and emergency medication available. Pre-medication with H1 antihistamine should be considered. If skin tests are positive, see the red zone.
Immediate systemic allergic reactions to drugs or vaccines	Specific risk allergy assessment concerning a possible PEG (or other excipients) allergy. If skin tests are negative, vaccination should be performed in a hospital setting with an observation of at least 60 minutes. If skin tests are positive, see the red zone.
**High risk**	
**Patient characteristics**	**Action**
- Positive skin tests to excipients - Previous allergic reaction to COVID-19 vaccine - Previous severe allergic reaction to a component of the vaccine or drugs, including PEG or trometamol	According to allergic risk assessment, the patient may be considered ineligible for a further vaccination or eligible for another vaccination, containing another excipient that resulted negative to the skin test. In all these cases, vaccination must be carried out under strict control in a hospital setting where emergency medical procedures for resuscitation are available.

### Final remarks

Even if the risk of anaphylaxis to COVID-19 mRNA vaccines, particularly to BNT162b2 vaccine, in children aged 5–11 years seems very low [4, 29], nevertheless a prudent approach still appears advisable as anaphylaxis is often unpredictable [13]. In case of previous systemic reaction to COVID-19 vaccines or drugs, including excipients contained in COVID-19 vaccines, an allergic evaluation is necessary, even if the evidence on the diagnostic utility of skin test for PEG or others excipients is still uncertain [5]. Risk assessment of allergic reactions to COVID-19 vaccines is useful to limit contraindications to vaccination and help to safely vaccinate people supposed to be at risk of allergic reactions [14]. All vaccine centers should follow national and international guidelines and have staff trained in recognizing and managing anaphylaxis [10, 13].

### COVID-19 vaccination in children with primary or acquired immunodeficiencies or history of multisystem inflammatory syndrome - children (MIS-C)/pediatric inflammatory multisystem syndrome temporally associated with SARS-CoV-2 (PIMS-TS)

Primary and acquired immunodeficiency syndromes encompass several heterogeneous conditions ranging from mild oligosymptomatic defects to severe diseases, with the phenotype of overwhelming opportunistic infections, autoinflammation, or immune dysregulation. Therefore, it is challenging to provide a “one-fits-all” recommendation for all children and adolescents affected by immunodeficiency syndromes. However, in general, no absolute contraindication exists to vaccination with approved mRNA or viral vector inactivated vaccines in individuals affected by primary or secondary immune defects [30, 31].

A consistent number of patients affected by inborn errors of immunity (IEI), including antibody deficiency syndromes under immunoglobulin replacement therapy, may mount an effective immune response against SARS-CoV-2 vaccines. Indeed, the type and strength of immune response could be very different accordingly to the type of IEI and the main immune compartment involved (i.e., the presence of a preserved adaptive B-cell and/or T-cell function) but also the patient’s therapy regimen under which the COVID-19 vaccine is administrated [31–34]. At the time of these recommendations, very little information exist about the efficacy of the anti-SARS-CoV-2 vaccine in patients affected by IEI, and consulting with a pediatric immunologist in most cases is advisable.

Very few data also exist about the risk/benefit ratio of anti-SARS-CoV-2 vaccination in children 5 to 11-years-old with previous MIS-C/PIMS-TS, although in theory even in these patients the benefits of COVID-19 vaccination outweigh the potential risks of triggering a relapse in the disease [34, 35]. However, in patients with MIS-C/PIMS-TS who have been treated with high-dose intravenous immunoglobulins (IVIG), it is suggested to start the vaccine schedule 6 months after IVIG administration, as well as full recovery and cardiac function normalization [34]. If untreated with IVIG, starting the COVID-19 vaccination may be considered 3 months after full recovery. Moreover, to the best of current knowledge, the next dose administration should be withheld in children who developed MIS-C/PIMS-TS after the COVID-19 vaccination. However, according to the available information, it is conceivable that these recommendations could change in the future.

## Conclusion

A teleconsulting visit with the referral pediatric immunologist is advisable in patients affected by IEI, acquired immunodeficiencies, and previous MIS-C/PIMS-TS.

## Data Availability

Data sharing is not applicable to this article as no datasets were generated or analysed during the current study.

## Abbreviations

PEG: polyethylene glycol
SIAIP: Società Italiana di Allergologia ed. Immunologia Pediatrica
SIAIC: Società Italiana Allergologia Immunologia Clinica
AAITO: Associazione Allergologi Italiani Territoriali Ospedalieri
MIS-C: Multisystem Inflammatory Syndrome - Children
PIMS-TS: Pediatric Inflammatory Multisystem Syndrome Temporally Associated With SARS-CoV-2
IEI: inborn errors of immunity
IVIG: intravenous immunoglobulins
